# Rapid Motion of the Blood

**Published:** 1893-01

**Authors:** 


### RAPID MOTION OF THE BLOOD.

Medical workers have made many curious experiments, but none more wonderful than that by which they ascertained the exact time required for the blood to make one entire trip through the system, which all students of physiology know means a complete circulation through the lungs, veins, arteries and the general capillary arrangements. Professors Dalton, Hering, Poisenille, Mattucci and Blake have been the chief investigators in this line, the first named having become more eminent in this particular branch of research from having the experience of others to fortify himself with.

All the old school anatomists believed that a considerable time elapsed, say from three to nine minutes, from the time when the blood left the right side of the heart, traversed the whole system, and then again returned to the starting point. Dalton has shown that the time is much shorter than was formerly generally supposed. The chief agent used in his experiments was a salt known to chemists as ferrocyanide of potassium, which can be readily detected in the blood on account of its chemical reaction. Professor Dalton describes the operation in the following language:

The blood was drawn from the jugular vein on the opposite side, and the interval which elapsed before the appearance of the foreign salt in the blood drawn from the second opening indicated the time required for the blood to pass from the point of injection through the vena cava to the heart, from the right side of the heart through the lungs to the left cavities, from the left ventricle through the carotid arteries and the capillary vessels of the head, and thence downward to the jugular vein in the opposite side. Dozens of carefully tabulated tests of this somewhat extraordinary subject show that the blood of man makes a complete circulation once every fifteen to twenty-five seconds, according to the physical condition of the subject experimented upon.

